# Development and Validation of Filters for the Retrieval of Studies of Clinical Examination From Medline

**DOI:** 10.2196/jmir.1826

**Published:** 2011-10-19

**Authors:** Nader Shaikh, Robert G Badgett, Mina Pi, Nancy L Wilczynski, K. Ann McKibbon, Andrea M Ketchum, R. Brian Haynes

**Affiliations:** ^1^University of Pittsburgh School of MedicineGeneral Academic PediatricsChildren’s Hospital of Pittsburgh of UPMCPittsburgh, PAUnited States; ^2^Department of Internal MedicineUniversity of Kansas School of Medicine at WichitaWichita, KSUnited States; ^3^Department of BiologyCarnegie Mellon UniversityPittsburgh, PAUnited States; ^4^Department of Clinical Epidemiology and BiostatisticsMcMaster UniversityHamilton, ONCanada; ^5^Health Sciences Library SystemUniversity of PittsburghPittsburgh, PAUnited States

**Keywords:** Medline, filter, hedge, clinical examination, recursive partitioning

## Abstract

**Background:**

Efficiently finding clinical examination studies—studies that quantify the value of symptoms and signs in the diagnosis of disease—is becoming increasingly difficult. Filters developed to retrieve studies of diagnosis from Medline lack specificity because they also retrieve large numbers of studies on the diagnostic value of imaging and laboratory tests.

**Objective:**

The objective was to develop filters for retrieving clinical examination studies from Medline.

**Methods:**

We developed filters in a training dataset and validated them in a testing database. We created the training database by hand searching 161 journals (n = 52,636 studies). We evaluated the recall and precision of 65 candidate single-term filters in identifying studies that reported the sensitivity and specificity of symptoms or signs in the training database. To identify best combinations of these search terms, we used recursive partitioning. The best-performing filters in the training database as well as 13 previously developed filters were evaluated in a testing database (n = 431,120 studies). We also examined the impact of examining reference lists of included articles on recall.

**Results:**

In the training database, the single-term filters with the highest recall (95%) and the highest precision (8.4%) were diagnosis[subheading] and “medical history taking”[MeSH], respectively. The multiple-term filter developed using recursive partitioning (the RP filter) had a recall of 100% and a precision of 89% in the training database. In the testing database, the Haynes-2004-Sensitive filter (recall 98%, precision 0.13%) and the RP filter (recall 89%, precision 0.52%) showed the best performance. The recall of these two filters increased to 99% and 94% respectively with review of the reference lists of the included articles.

**Conclusions:**

Recursive partitioning appears to be a useful method of developing search filters. The empirical search filters proposed here can assist in the retrieval of clinical examination studies from Medline; however, because of the low precision of the search strategies, retrieving relevant studies remains challenging. Improving precision may require systematic changes in the tagging of articles by the National Library of Medicine.

## Introduction

In arriving at a diagnosis, clinicians often rely on clinical examination findings (ie, information from the patient’s history and/or physical examination) [1-3]. Therefore, easy availability of results from clinical examination studies can greatly influence medical care. The number of studies published per year that focus on clinical examination has more than tripled since 1980. As this literature multiplies, the task of reliably and simply identifying sound studies is becoming increasingly challenging.

In many areas of medicine, filters have been developed to facilitate the search for relevant articles. Filters are pretested search strategies that help identify studies of a certain type from among all the other studies in Medline. Search filters that are optimized for the retrieval of studies of diagnosis, therapy, and clinical prediction rules are available [4-6]. These filters are routinely used by both clinicians (eg, PubMed Clinical Queries [4]) and systematic reviewers (eg, Cochrane Highly Sensitive Search Strategy for therapy articles [7]). No published filters, however, have been developed to facilitate the search for studies of clinical examination [8]. A clinical examination filter may be useful to clinicians and authors of systematic reviews. Clinicians need to identify sound clinical examination articles in a timely fashion so that they can effectively care for their patients. With the commencement of Cochrane reviews of Diagnostic Test Accuracy [9], which will include reviews of clinical examination, there is a growing need for filters optimized for the retrieval of clinical examination studies.

The goal of this study was to develop and evaluate Medline filters that could facilitate retrieval of clinical examination studies.

## Methods

### Overview

The training and testing of the filters entailed 8 steps: (1) development of a training database, (2) identification of candidate single-term filters, (3) identification of single-term filters with the best performance in the training database, (4) identification of the multiple-term filter with the best performance in the training database using recursive partitioning, (5) development of a testing database, (6) evaluation of the performance of filters developed in this study in the testing database, (7) evaluation of the performance of previously developed filters in the testing database, and (8) examination of the impact of reviewing reference lists of included articles on recall. We performed our research using PubMed, the United States National Library of Medicine’s public search engine for accessing Medline.

### Development of a Training Database

We used the Clinical Hedges database, the methods of which have been previously described [10], as the starting point for this study. Briefly, the Hedges team conducted a hand search of articles published in the year 2000 in 161 prominent journals that met criteria for high quality; a total of 52,636 articles were reviewed. The team categorized articles as pertaining to diagnosis, therapy, or prognosis (among other categories) based on a priori criteria. For the project reported here, we reviewed the studies identified in the Clinical Hedges database as pertaining to diagnosis or prognosis to identify those that specifically pertained to clinical examination (n = 1347).

**Figure 1:**
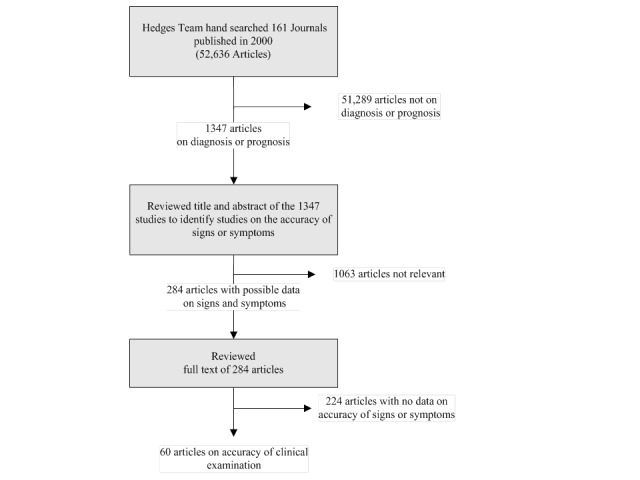
Flow sheet describing development of the training database.

One investigator (author NS) initially reviewed the title and abstract (if an abstract was available) and full text, if necessary, of the 1347 studies and classified each article as a *clinical examination* (gold standard article) or a *non* *–* *clinical examination* article (Figure 1). Gold standard articles were those that met our a priori criteria for quantifying the value of the clinical examination. We only considered physical examination findings that could be elicited with commonly available props such as a stethoscope or ophthalmoscope. We included articles that reported both sensitivity and specificity for at least one symptom, sign, or a combination of signs and symptoms (Figure 1). We included multivariable diagnostic rules if they were composed of only signs or symptoms; studies describing a multivariable rule that included imaging or laboratory findings were not considered because these studies can easily be found using existing, more general filters designed for the detection of diagnostic tests. For example, the Breese score—a validated scoring system to diagnose streptococcal pharyngitis in children—was not considered a clinical examination study because, in addition to signs and symptoms, a white blood cell count is required to calculate a total score [11]. We excluded studies of prognostic factors, that is, those focusing on the prediction of future disease (eg, prediction of mortality based on findings on admission to the intensive care unit). Articles with less than 10 patients were excluded because these studies, due to their very small sample size, cannot provide accurate estimates of sensitivity or specificity. Studies that could not be easily categorized were independently reviewed by a second reviewer (author RGB) and differences were resolved by discussion. This process resulted in 60 of 52,636 articles meeting the gold standard criteria (Figure 1).

We then recreated the Clinical Hedges dataset by entering the 161 journals in Medline and by restricting the publication year to 2000 (Figure 1). We placed the articles into two collections stored in an account at PubMed. One collection contained the articles that met our criteria for gold standard, and the other collection contained the remaining articles. 

### Identification of Candidate Single-Term Filters

We generated a list of 65 candidate search terms in PubMed syntax with the help of two clinicians, three reference librarians, and a thorough review of the literature. The expert searchers independently reviewed our lists of candidate terms and suggested additional terms. We used terms pertaining to clinical examination and diagnosis as well as negated terms (eg, NOT MRI). (See Multimedia Appendix 1 for a list of the search terms used.) The following PubMed syntax was used: [tw] = text word; [MeSH] = National Library of Medicine's Medical Subject Heading; [sh] = MeSH subheading; [TIAB] = Title or abstract; [pt] = publication type; [ti] = title; du[sh] = diagnostic use MeSH subheading; noexp = do not explode (ie, do not automatically include the more specific terms beneath the MeSH term in the MeSH hierarchy).

### Identification of Best-Performing Single Term Filters Using the Training Database

We evaluated each individual filter against the training database to determine its recall (proportion of the clinical examination articles that the filter detected), precision (proportion of articles retrieved that were relevant), F-measure (an overall measure combining recall and precision), “fallout” (the proportion of nonrelevant articles that were retrieved), and the number needed to read (the average number of articles the searcher will need to look at to find each relevant article) [12]. Of the clinical examination terms, 7 had a recall of greater than 25% and a fallout of less than 50%. We evaluated all possible combinations (2-term combinations, 3-term combinations, 4-term combinations, 5-term combinations, 6-term combinations, and one 7-term combination) of these 7 terms to identify the combinations with the highest recall, precision, and F-measure. We repeated this process for all possible combination of the 8 diagnosis terms with a recall of greater than 25% and a fallout of less than 50%.

### Development of a Multiple-Term Filter Using Recursive Partitioning Using the Training Database

Because testing all combinations of single-term filters would have been prohibitive, we used recursive partitioning to develop the best multiple term filter (hereinafter referred to as the recursive partitioning filter) [13]. Recursive partitioning is a form of nonparametric discriminant analysis that repeatedly stratifies the group into smaller mutually exclusive subgroups according to a set of predictor variables. Apart from its efficiency, an added advantage of recursive partitioning is its ability to create filters including both Boolean terms, OR and AND. Recursive partitioning also adds the ability to vary misclassification costs (cost of a false positive vs costs of a false negative) in order to identify terms that best address the objectives of the analysis. For each of the 41 terms with a recall of greater than 25% *or* fallout less than 75%, we calculated the recall, precision, F-measure, and fallout against the training database. To decide on the first branching point in the tree, we chose the term with the lowest weighted error rate (weight based on the prevalence of clinical examination studies among all studies in the database) [13]. Once the term with the lowest error rate was found (ie, diagnosis[tw]), we created 4 new datasets in PubMed (“clinical examination” AND diagnosis[tw]; “clinical exam” NOT diagnosis[tw]; “non clinical examination” AND diagnosis[tw]; “Non clinical exam” NOT diagnosis[tw]). We then tested all remaining filters against each of these 4 new datasets and again identified terms with the lowest error rate. This allowed us to grow the recursive partitioning tree. We repeated this until the two 2x2 tables created by the split were no longer significantly different from each other (*P* > .05). Because this approach can lead to overfitting, we also required each new branch to have a recall of at least 99%.

### Establishing the Testing Database

**Figure 2:**
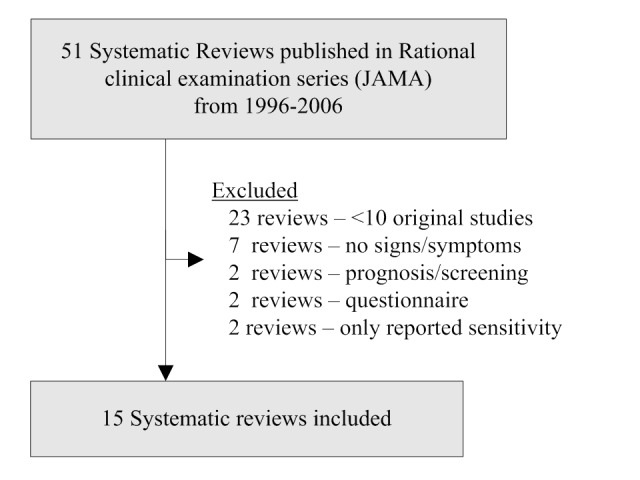
Flow sheet describing development of the testing database.

To develop the testing database, we used the largest collection of systematic reviews on clinical examination in the literature: The Rational Clinical Examination series in the Journal of the American Medical Association (JAMA) [14]. One author (NS) used a priori inclusion and exclusion criteria similar to those used to establish the training database to develop the testing database. We included systematic reviews that reviewed at least 10 original studies, reported sensitivity and specificity of signs or symptoms, and were published beginning in 1996 through 2006 (Figure 2). Reviews that did not pertain to clinical examination, reviews of questionnaires, reviews of multivariable diagnostic rules that included laboratory or imaging variables, and reviews of prognostic or screening tests were excluded. A total of 15 systematic reviews met all inclusion criteria.

Articles included in these 15 reviews were regarded as *relevant* articles (gold standard) that the filters should be able to recall. To identify *nonrelevant* articles we recreated the subject-specific search (eg, temporal arteritis or giant cell arteritis) using the search strategy reported in the methods section of each of the systematic reviews; articles that were retrieved by the electronic subject-specific search but that were not included in the review were regarded as nonrelevant. This allowed us to calculate the number of relevant and nonrelevant articles for each review. A total of 224 original clinical examination articles were included in these 15 systematic reviews. We excluded 7 articles that were not in Medline. One study was excluded because it was not found by the subject-specific search. In all, 28 older studies without abstracts were excluded because filters would have difficulty retrieving these studies and because contemporary studies of the clinical examination are likely to have abstracts. The resulting testing database included 188 articles that were relevant and 430,932 articles that were nonrelevant. 

### Evaluation of the Filters Developed in This Study

For the 3 filters with the highest recall in the training database, we calculated the recall, precision, F-measure, and the number needed to read in the testing database. The calculations were based on the cells and formulas in Table 1.

**Table 1:** A 2x2 table created for each systematic review and formulas used^a^

	Articles Included in the Systematic Review	Articles Not Included in the Systematic Review
Detected by filter	A	B
Missed by filter	C	D

^a^ Recall = A/(A+C); Precision = A/(A+B); F-measure = 2*precision*recall/(precision + recall); Number needed to read = 1/precision; Fallout = B/(B+D) [15,16]

### Evaluation of Previously Developed Filters

The performance of 12 previously developed filters validated for retrieving articles on diagnosis [10,17-21] and 1 filter developed specifically for the clinical examination by editors of the Rational Clinical Examination series [22] was evaluated in the testing database. The filters tested are listed in Multimedia Appendix 2 and are named using the name of the first author of the publication describing the filter followed by the year of publication. If more than one filter was described in the publication, we tagged on the name used by the author to describe the various filters. For example, the label “Haynes-2004-Sensitive” refers to the filter described by Haynes et al in their 2004 publication with the highest sensitivity (ie, the filter with the highest recall). Finally, we tested whether a combination of the best filters would improve performance.

### Impact of Reviewing Reference Lists on Recall

Authors of systematic reviews often examine reference lists hoping to increase recall. We examined how this strategy would complement the use of filters in the area of clinical examination. Specifically, we examined whether checking the reference lists of included articles would allow use of a filter with a lower recall. Thus, we identified articles that were missed by the 2 filters with the highest recall and checked to see if these articles were included in the reference lists of the articles not missed by these filters.

## Results

### Training Results

Filters with the best performance in the training database are shown in Table 2. The term diagnosis[subheading] identified 95% of the clinical examination studies. The MeSH term physical examination identified only 25% of studies and was therefore not included in the table. In general, multiple-term search filters using only terms pertaining to diagnosis outperformed the filters using only clinical examination terms. Also, 3 filters had a recall of 100% (CE-high recall, Dx-high recall, RP) and two of these (Dx-high recall, RP) appeared particularly promising because of their higher precision.

**Table 2:** Filters with the best recall (keeping fallout less than 50%), precision (keeping recall greater than 50%) and F-measure in the training database

Filter		Performance Measure	Recall (%)	Precision (%)	F-measure	NNR^a^
**Best single-term filter**						
	Diagnosis[subheading]	Best recall	95	0.35	0.71	279
	Medical history taking[MeSH]	Best precision and F-measure	12	8.44	9.79	11.86
**Best multiple-term filters using only diagnosis terms**						
	Diagnosis[tw] OR "sensitivity and specificity"[MeSH]	Best recall (hereinafter Dx-high recall)	100	0.52	1.04	191
	Predictive value of tests[mesh] OR specificity[TIAB]	Best precision and F-measure (hereinafter Dx-precise)	67	1.95	3.78	51
**Best multiple-term filters using only clinical examination terms**						
	Clinical*[tw] OR symptom*[tw] OR exam*[tw] OR criteria[tw] OR tests[tw] OR test[tw]	Best recall (hereinafter CE-high recall)	100	0.27	0.53	377
	Tests[tw] OR physical[tw]	Best precision and F-measure (hereinafter CE-precise)	62	0.72	1.43	138
**Best multiple-term filter using all terms**						
	(Diagnosis[tw] AND (specific*[tw] OR clinical*[tw] OR exam*[tw])) OR "sensitivity and specificity"[MeSH]	Best overall filter from recursive partition (hereinafter RP-filter)^b^	100	0.89	1.76	113

^a^ Number needed to read

^b^Filter developed using recursive partitioning (see “Methods” section)

**Figure 3:**
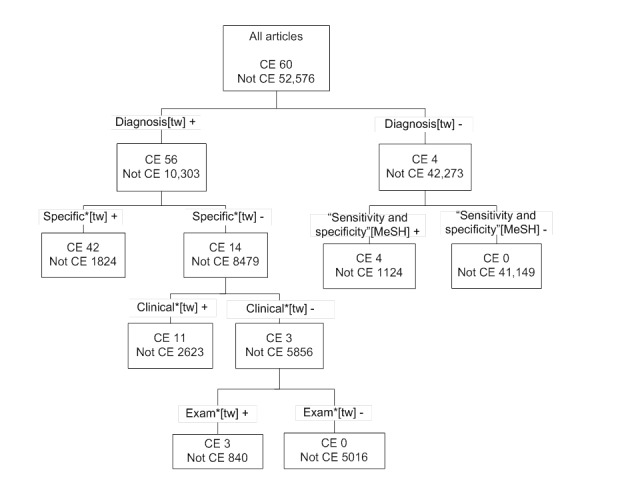
Best multiple-term filter for retrieval of articles on clinical examination (CE) developed using recursive partitioning.

The recursive partitioning tree is shown in Figure 3. When converted to Boolean language, the RP filter, in PubMed syntax, is as follows: (Diagnosis[tw] AND (specific*[tw] OR clinical*[tw] OR exam*[tw])) OR "sensitivity and specificity"[MeSH].

### Testing Results

The recall, precision, F-measure, and the number needed to read for the filters developed in this study as well as the 13 previously developed filters and combination of filters are presented in Table 3. The Haynes-2004-Sensitive filter[10] had the highest recall (98%). When considering only filters with a recall of 80%, the RP filter had the highest precision (0.26%). The Haynes-2004-Sensitive filter and the CE-high recall filter when combined using the Boolean term OR had a recall of 100% and a precision of 0.06%. Other filter combinations did not offer much of an improvement in recall compared with their individual use.

**Table 3:** Performance of the search filters in the testing database sorted according to recall

Filters or Filter Combinations		Recall (%)	Precision (%)	F-measure	NNR^a^
**Filters**					
	Haynes-2004-Sensitive [10]	98	0.13	0.26	778
	Vincent-2003 [21]	98	0.09	0.17	1154
	Bachmann-2002 [15]	96	0.11	0.22	906
	Haynes-1994-Sensitive [19]	95	0.16	0.31	641
	Dx-high recall^b^	95	0.12	0.25	804
	Van der Weijden-1997 [20]	95	0.07	0.13	1490
	CE-high recall^b^	91	0.08	0.15	1330
	Haynes-1994-Accurate [19]	91	0.07	0.14	1431
	RP-filter^b^	89	0.26	0.52	380
	Rational Clinical exam [22]	73	0.30	0.61	328
	Deville-2002 [18]	71	0.40	0.80	249
	Haynes-2004-Accurate [10]	69	0.45	0.89	224
	Deville-2000-Accurate [17]	64	0.64	1.26	157
	Deville-2000-Sensitive [17]	64	0.60	1.19	167
	Haynes-1994-Specific [19]	51	0.72	1.42	139
	Haynes-2004-Specific [10]	36	1.01	1.97	99
**Filter combinations**					
	Haynes-2004-Sensitive [10] OR CE-high recall	100	0.06	0.12	1613
	CE-high recall OR RP	99	0.06	0.13	1572
	Haynes-2004-Sensitive [10] OR RP	98	0.11	0.22	890
	Haynes-2004-Sensitive [10] AND RP	95	0.13	0.25	790
	Haynes-2004-Sensitive [10] AND CE-high recall	88	0.19	0.39	515

^a^NNR = number needed to read

^b^The three filters with highest recall in the training database

### Impact of Reviewing Reference Lists

Overall, 4 of 188 relevant articles were missed by the Haynes-2004-Sensitive search strategy, and, of these, 2 were retrieved by reviewing the reference lists of the articles not missed by this strategy (increasing recall from 98% to 99%). Of the 19 articles missed by the recursive partitioning strategy, 8 were retrieved by reviewing the reference lists of the articles not missed by this strategy (increasing recall from 89% to 94%).

## Discussion

We quantified the recall and precision of filters that may be used to find articles on clinical examination in MEDLINE. While the use of recursive partitioning may increase the precision of searching, all of the strategies we tested had a very low precision of less than 2%.

### Application of the Filters

For health care providers looking for information regarding the diagnostic accuracy of clinical examination findings, the RP filter appears to be the most reasonable choice. For example, let us assume that a clinician is reviewing the ability of the third heart sound to detect heart failure. To determine the posttest probability of congestive heart failure among patients with a third heart sound, the search using the RP filter in PubMed would be (gallop OR S3 OR third heart sound) AND heart failure[MeSH] AND ((Diagnosis[tw] AND (specific*[tw] OR clinical*[tw] OR exam*[tw])) OR "sensitivity and specificity"[MeSH]). As of March 2011 this search yielded 68 articles, several of which directly related to the clinician’s question. Although not studied, the physician could restrict the search to systematic reviews by adding the term “systematic[sb]”. This strategy yielded 1 relevant systematic review. While the NNRs for the filters examined reported in this study are very high (Table 3), the NNR will be considerably lower in clinical practice. The NNR, like the positive predictive value of a diagnostic test, is dependent on the prevalence of articles about physical examination. Although the proportion of physical examination studies in MEDLINE is relatively low (eg, < 0.1% in the Hedges database), when the clinician enters search terms for a disease and for the physical examination findings, the prevalence of physical examination articles increases. As a result, the number needed to read will be substantially lower (see example above). Accordingly, it is critical that the clinician uses a well-built clinical question using the most descriptive and specific terms possible [23].

For the researcher who wants to undertake a systematic review, the Haynes-2004-Sensitive filter [10], with its 98% recall, appears to be the most reasonable choice. Nevertheless, some articles may be missed if one relies on this filter alone. Two strategies are suggested for increasing recall. One is to examine the reference list of the articles meeting criteria for inclusion. This increases the sensitivity to 99%. The other strategy is to combine the Haynes-1994-and the CE High recall filter using OR. Although this strategy had a 100% recall, its precision was very low (0.06%). Even though relying on filters alone may lead to some studies being missed [24], we feel that use of filters is appropriate, especially when it is exceedingly difficult to conduct a review without one. The filters presented here are intended to be used as part of a larger search strategy, which includes a review of reference lists, and communication with experts in the field.

### Poor Precision of Filters for Clinical Examination Studies

All of the filters we tested had a very low precision in identifying clinical examination studies. Our findings are consistent with findings published by Haynes and colleagues [19], indicating poor precision of filters developed for retrieving articles on diagnosis as compared to those developed for retrieving articles on treatment (Table 4). These observations suggest that the National Library of Medicine should create a publication type for studies that quantify sensitivity and specificity for diagnosis. Other alternative or complementary solutions may involve manually identifying and tagging studies that quantify the clinical examination (as is currently used by the Cochrane Collaboration to create a database of sound randomized controlled treatment trials, CENTRAL), collaborative filtering, or content-based filtering [25].

**Table 4:** Comparison of the performance of filters for clinical examination, diagnosis, and treatment

Filters		Recall (%)	Precision (%)	F-measure	NNR^a^
**Clinical examination**					
	Haynes-2004-Sensitive [10]	98	0.13	0.26	778
	Recursive partitioning	89	0.26	0.52	380
**Diagnosis in general**					
	Haynes-2004-Sensitive [10]	99	1.1	2.17	91
**Treatment**					
	Haynes 2005 [26]^b^	99	9.9	18.0	10
	Haynes 1994 [19]^b^	99	22	36.0	4.5

^a^NNR = Number needed to read

^b^Values are for the most-sensitive multi-term filter

### Limitations

There are several limitations to our study. The Hedges database [10] contains the 161 journals whose articles were felt to have the highest scientific merit and clinical relevance. While we believe these are the journals that will most help clinicians, the results may vary when all of Medline is searched. In addition, journals published in foreign languages were not included in the Hedges database. Because some of our filters used text words, these filters may fall in performance when searching for articles that have not been translated to English or for articles without an abstract. Another limitation was in our identification of candidate search terms. Consistent with prior studies of filter development, expert searchers independently reviewed our lists of candidate terms and suggested additional terms. However, we did not quantitatively review the most frequent search terms and text words in the gold standard studies to identify candidate terms. However, when we retrospectively examined the MeSH terms that were used to index the gold standard studies in the training database, the terms not tested by us had substantially lower recall and precision than the terms we selected. Nevertheless, we believe that future studies should incorporate this method of identifying terms. Another limitation was in identification of the gold standard articles in the training database. Only one investigator initially reviewed the articles for eligibility. Future studies should utilize two investigators who independently assess each article. Finally, because of the low prevalence of clinical examination studies, the number of gold standard studies in both the training and testing databases were relatively small. Further testing of these filters in larger databases is necessary.

A surprising result is that only 25% and 20% of the clinical examination studies in the training database were coded with the MeSH terms “physical examination” and “signs and symptoms”, respectively. This current inconsistency in the assignment of these MeSH terms limits the ability of search filters on this topic.

### Implications for Future Filter Development

We present a new method for the development of multi-term filters. The use of recursive partitioning in the development of filters is novel and seems particularly well suited when there are many candidate terms. When the number of candidate terms is small, one could test all the possible combinations of terms against the dataset. This becomes prohibitive when the number of candidate terms is large. In contrast, using recursive partitioning, a search filter is constructed in a stepwise fashion. This method also allows for the development of filters that use both AND and OR terms and allows for the development of filters with the best combination of recall and precision.

### Conclusions

Recursive partitioning offers an alternative method of developing filters: it not only allows for the development of filters with the best combination of recall and precision, but also for the development of filters that use both AND and OR Boolean connectors. Despite the advantages of recursive partitioning, the filters we developed for the retrieval of clinical examination studies had relatively low precision. We believe the National Library of Medicine should create a publication type for articles that quantify the sensitivity and specificity of the clinical examination. This new tag could improve retrieval of studies of clinical diagnosis.

## Multimedia Appendix 1

List of single-term filters.

## Multimedia Appendix 2

List of filters evaluated in the testing corpus.
