# Correction: Development and Validation of Filters for the Retrieval of Studies of Clinical Examination From Medline

**DOI:** 10.2196/jmir.2232

**Published:** 2012-08-03

**Authors:** Robert Badgett, Nader Shaikh, Mini Pi, Nancy L Wilczynski, K. Ann McKibbon, Andrea M Ketchum, R. Brian Haynes

**Affiliations:** ^1^University of Kansas School of Medicine at WichitaDepartment of Internal Medicine and Department of Preventive Medicine and Public HealthWichita, KSUnited States; ^2^Children’s Hospital of Pittsburgh of UPMCGeneral Academic PediatricsUPMCPittsburgh, PAUnited States; ^3^Carnegie Mellon UniversityDepartment of BiologyPittsburgh, PAUnited States; ^4^McMaster UniversityDepartment of Clinical Epidemiology and BiostatisticsHamilton, ONCanada; ^5^University of PittsburghHealth Sciences Library SystemPittsburgh, PAUnited States

**Keywords:** information retrieval, MEDLINE

The authors of the recent JMIR publication “Development and Validation of Filters for the Retrieval of Studies of Clinical Examination From Medline” (J Med Internet Res 2011;13(4):e82) [1] inadvertently misplaced a decimal point in the final row of Table 4 (Comparison of the performance of filters for clinical examination, diagnosis, and treatment). The precision of the original report of the Haynes 1994 filter should be 22% and not 0.22. This leads to the F-measure being 36 and the NNR being 4.5. In addition, the following line in the discussion is incorrect and has been removed: “In addition, our results are consistent with Haynes initial reports of locating trials in the early 1990s (19) before the National Library of Medicine collaborated with the Cochrane Collaboration and introduced 'randomized controlled trial' as a MeSH term (final row of Table 4)”.

The data in Table 4 was compiled to support our recommendation that the National Library of Medicine should create a publication type for “diagnostic accuracy study” to label studies that quantify sensitivity and specificity for diagnosis. We regret the error, but do not believe it weakens our recommendation or alters the results of our study.

The precision of the Haynes 1994 treatment filter was surprisingly high in their study and is due in part to the development and validation being limited to 10 high-impact journals [2]. Our study used 161 journals.

The online version of the JMIR paper has been corrected together with publication of this correction notice. A corrected version has been submitted by the publisher to PubMed Central, but incorrect versions may persist on other sites.
