# Supplementary material for: Development and Validation of Filters for the Retrieval of Studies of Clinical Examination From Medline
Source: J Med Internet Res. 2011 Oct 19;13(4):e82. doi: 10.2196/jmir.1826 (PMC3222198; doi:10.2196/jmir.1826)
Supplement: Supplementary file 1 [file jmir_v13i4e82_app1.pdf]

Physical examination terms	Diagnosis terms	Other terms
Clinical[tw]*	*Predictive value of tests[MeSH]*	*hasabstract*
Symptom[tw]*	*"Sensitivity and specificity"[MeSH]*	*NOT ultraso*[ti]*
Exam[tw]*	*Predict*[tw]*	*NOT MRI[ti]*
Criteria[tw]	*Diagnostic * [MeSH]*	*NOT magnetic reson*[ti]*
Finding[tw]*	*Specificity[TIAB]*	*NOT CT[ti]*
Tests[tw]	*Specific*[tw]*	*NOT tomogr*[ti]*
Test[tw]	*Diagnosis[tw]*	*NOT assay*[ti]*
Physical[tw]	*Diagnosis[sh:noexp]*	*NOT scintig*[ti]*
Signs and Symptoms[MeSH]	Variable*[tw]	*NOT radiog[ti]*
Physical Examination[MeSH]	Testing[tw]	*NOT radionuc*[ti]*
Histor[tw]*	*Diagnosis[sh]*	*NOT Editorial[pt]*
Sign[tw]	*Diagnos*[tw]*	*NOT Letter[pt]*
Signs[tw]	*Diagnosis[MeSH]*	*NOT Practice Guideline[pt]*
Physical exam*[tw]	Du[sh]	*NOT Randomized Controlled Trial[pt]*
Feature*[tw]	Diagnostic* [MESH:noexp]	*NOT Addresses[pt]*
Auscultat*[tw]	"Diagnosis, differential"[MESH]	*NOT Bibliography[pt]*
Palpa*[tw]	Post-test probab*[tw]	*NOT Biography[pt]*
Percuss*[tw]	Pre-test probab*[tw]	*NOT Comment[pt]*
Inspect*[tw]	Likelihood ratio*[tw]	*NOT review[pt]*
Medical History Taking[MeSH]	ROC[tw]	
	Bayes theorem[tw]	
	Diagnostic accuracy[tw]	
	Correlat*[tw]	
	Associat[tw]*	
	Relat*[tw]	
	Marker [tw]	

[a]PubMed uses the asterisk * as its truncation symbol.

[b]Terms used in recursive partition model are italicized.

[c] [tw] = text word; [MeSH] = National Library of Medicine's Medical Subject Heading; [sh] = MeSH subheading; [TIAB] = Title or abstract; [pt] = publication type; [ti] = title; du[sh] = diagnostic use MeSH subheading; noexp = do not explode
