# Supplementary material for: Development and Validation of Filters for the Retrieval of Studies of Clinical Examination From Medline
Source: J Med Internet Res. 2011 Oct 19;13(4):e82. doi: 10.2196/jmir.1826 (PMC3222198; doi:10.2196/jmir.1826)
Supplement: Supplementary file 2 [file jmir_v13i4e82_app2.pdf]

Filter	Search Filters (shown in PubMed syntax)
RP[†]	(Diagnosis[tw] AND (specific*[tw] OR clinical*[tw] OR exam*[tw])) OR "sensitivity and specificity"[MESH]
Dx-high recall[†]	Diagnosis[tw] OR "sensitivity and specificity"[MeSH]
CE-high recall[†]	Clinical*[tw] OR symptom*[tw] OR exam*[tw] OR criteria[tw] OR tests[tw] OR test[tw]
Haynes-1994-Sensitive[19]	“sensitivity and specificity”[MeSH] OR diagnosis[sh:noexp] OR “diagnostic use”[sh] OR sensitivity[tw] OR specificity[tw]
Haynes-1994-Specific[19]	“sensitivity and specificity”[MeSH] OR (predictive[tw] AND value[tw])
Haynes-1994-Accurate[19]	“sensitivity and specificity”[MeSH] OR diagnosis[MeSH] OR “diagnostic use”[sh] OR sensitivity[tw] OR (predictive[tw] AND value[tw])
Van der Weijden-1997[20]	“Diagnosis”[MeSH] OR “sensitivity and specificity”[MeSH] OR “Reference values”[MeSH] OR “False positive reactions”[MeSH] OR “False negative reactions”[MeSH] OR “Mass screening”[MeSH] OR diagnos* OR sensitivity OR specificity OR predictive value* OR reference value* OR ROC* OR likelihood ratio* OR monitoring
Deville-2000-Accurate[17]	“sensitivity and specificity”[MeSH] OR specificity[tw] OR false negative[tw] OR accuracy[tw]
Deville-2000-Sensitive[17]	“sensitivity and specificity”[MeSH] OR specificity[tw] OR false negative[tw] OR screening[tw]
Bachmann-2002[15]	“sensitivity and specificity”[MeSH] OR predict* OR diagnose* OR diagnosi* OR diagnost* OR accura*
Vincent-2003[21]	“sensitivity and specificity”[MeSH] OR sensitivity[tw] OR specificity[tw] OR accuracy[tw] OR predictive value*[tw] OR ROC curve*[tw] OR false positive*[tw] OR false negative*[tw] OR observer variation*[tw] OR likelihood ratio*[tw] OR “Likelihood functions”[MeSH] OR “mass screening”[MeSH] OR "diagnosis,differential”[MeSH] OR Diagnostic errors[MeSH] OR diagnosis[sh] OR “diagnostic use”[sh:noexp]
Haynes-2004-Sensitive[10]	Sensitiv*[Title/Abstract] OR sensitivity and specificity[MeSH] OR diagnos*[Title/Abstract] OR diagnosis[MeSH:noexp] OR diagnostic*[MeSH:noexp] OR diagnosis,differential[MeSH:noexp] OR diagnosis[sh:noexp]
Haynes-2004-Specific[10]	Specificity[tw]
Haynes-2004-Accurate[10]	Sensitiv* OR predictive value* OR accuracy[tw]
Deville-2002[18]	Specificity[tw] OR screening[tw] OR accuracy OR predictive value* OR ROC OR likelihood ratio[tw] OR false positive OR false negative OR “sensitivity and specificity” OR predictive value OR reference value*
Rational Clinical Exam[22]	“Physical examination”[MeSH] or physical exam* OR “medical history taking”[MeSH] OR “professional competence”[MeSH] OR “sensitivity and specificity” OR "sensitivity and specificity"[MeSH] OR “reproducibility of results”[MeSH] OR “observer variation”[MeSH] OR “diagnostic tests, routine”[MeSH] or “decision support techniques”[MeSH] OR “Bayes theorem”[MeSH]

[†]Developed in this manuscript
